# Long-term psychological and neurological outcomes among people with a history of non-malignant meningioma in the UK Biobank cohort

**DOI:** 10.1093/noajnl/vdaf105

**Published:** 2025-06-05

**Authors:** Diana R Withrow, Fahad S Al-Huda, Helen Bulbeck, Krishnan Bhaskaran, Robin Grant, Usama Ali, Pieter Pretorius, Jason L Oke, Brian D Nicholson

**Affiliations:** Nuffield Department of Primary Care Health Sciences, University of Oxford, Oxford; Clinical Effectiveness Unit, Royal College of Surgeons of England, London; Clinical Effectiveness Unit, Faculty of Public Health and Policy, London School of Hygiene and Tropical Medicine, London; Nuffield Department of Primary Care Health Sciences, University of Oxford, Oxford; brainstrust, Cowes, Isle of Wight; Department of Non-Communicable Disease Epidemiology, Faculty of Epidemiology and Population Health, London School of Hygiene and Tropical Medicine, London; Department of Clinical Neurosciences, Royal Infirmary of Edinburgh, Edinburgh; Nuffield Department of Population Health, University of Oxford, Oxford; Department of Neuroradiology, John Radcliffe Hospital, Oxford University Hospitals NHS Foundation Trust; Abbott Diabetes Care, Witney, Oxfordshire; Nuffield Department of Primary Care Health Sciences, University of Oxford, Oxford

**Keywords:** brain tumours, meningioma, late effects, survivorship

## Abstract

**Background:**

Meningiomas are among the most common brain tumours in the United Kingdom and incidence is increasing. Up to 90% of meningiomas are Grade 1 (non-malignant) and have high survival. Brain tumour survivors are at risk of psychological and neurological late effects but risks in persons with non-malignant meningioma are not well characterized.

**Methods:**

We used UK Biobank, a cohort of approximately 500 000 adults recruited ages 40-69 during 2006-2010. Non-malignant meningioma patients were identified through linked cancer registry data. Follow-up for 10 outcomes was based on linkage to Hospital Episode Statistics. Standardized incidence ratios (SIRs) compared risks in meningioma patients to rates from UK Biobank overall, adjusted for age, sex, and calendar time.

**Results:**

Four hundred and sixty-seven individuals were diagnosed with non-malignant meningioma after joining UK Biobank (77% female). Median age at diagnosis was 65 and median follow-up was 5 years. Persons with meningioma had significantly increased risks of 9 of 10 sequelae studied. The lowest SIR was for stroke (1.3, 95% confidence interval [CI] 0.6-2.9) and the highest SIRs were for epilepsy (18.9, 95%CI: 13.3-26.9) and visual disturbances (6.6, 95%CI: 3.3-13.1). SIRs for depression, anxiety, headache, fatigue, hearing loss, limb weakness, and cognitive issues (in ascending order) ranged from 2.3 (95%CI: 1.6-3.4) to 4.4 (95%CI: 2.8-6.9).

**Conclusions:**

By providing preliminary evidence of excess risk of a range of long-term sequelae, this research can provide insight into future risks and validate survivor experience for people diagnosed with non-malignant meningioma and build momentum for future research using larger population-based databases and primary care records.

**Key Points**

- Meningiomas are common brain tumours with rising incidence rates.
- Electronic medical record linkage in a large cohort study examined long-term outcomes.
- Results show increased risks of psychological and neurological sequelae in survivors.

**Importance of the Study**

Meningiomas are among the most common brain tumours, with rising incidence rates. The long-term effects of non-malignant meningioma have not been thoroughly described. This study is the first to examine long-term neurological and psychological outcomes in individuals with non-malignant meningioma using the UK Biobank. By using linked electronic health records, the study provides the largest analysis of its kind. The findings confirm increased risks for several health issues, including epilepsy and visual disturbances, validating patient concerns. This study underscores the need for more research to enhance the understanding and management of non-malignant meningioma in the years after a diagnosis.

Meningiomas are tumours that occur in the tissue covering the brain and spinal cord (meninges) and are the most frequently diagnosed brain tumours among women, and the second most common brain tumour among men in the United Kingdom.^1^ Grade 1 (non-malignant) meningiomas are slow growing, localized, and can often be treated with observation or surgery alone, whereas Grade 2 (atypical) or 3 (malignant) meningiomas can spread to surrounding tissues, often requiring more aggressive treatment. Up to 90% of meningiomas are Grade 1.^2^ The incidence of Grade 1 meningiomas has been increasing in England by 8% per year on average since the early 1990s.^3^ Increases have also been recorded in the United States^4^ likely owing at least in part to advancements in diagnostic techniques which have increased detection rates.^1,5^

These Grade 1 meningiomas (henceforth referred to as non-malignant meningiomas [NMMs]) are not classified as cancer but depending on their location and size, they may cause serious symptoms. For symptomatic and/or growing NMMs, the primary treatment is surgery with the goal of relieving symptoms. Stereotactic radiosurgery and fractionated radiotherapy can be indicated in some cases but are most often reserved for higher grade (WHO grade 2 or 3) tumours.^6^ In a US study of nearly 50 000 meningioma patients, of whom 94% had non-malignant histology, over 40% underwent surgery, a likely underestimate of the true proportion of patients undergoing surgery as the study was registry-based.^7^ The long-term consequences of NMM are not well understood as people with NMM have seldom been studied from a long-term survivorship perspective despite the likely neurological and psychological risks associated with the surgery and the tumour itself.

Much of the research to date has focussed on brain tumours broadly,^8–12^ or, when studying NMM, have had short-term follow-up of around 1 year after diagnosis.^13,14^ A few studies have focussed specifically on people with a history of NMM and have had longer follow-up. One Swedish study of 190 patients with median follow-up of 9 years reported that 49% of patients had impaired health-related quality of life (HRQOL), and 43% suffered from neurocognitive deficits.^15^ In a study carried out in Melbourne, 291 patients followed up for up to 10 years self-reported decreased HRQOL across a number of domains compared to a healthy reference.^16^ In a study carried out in the Netherlands, among 89 patients with 11.5 years of median follow-up, 67% showed at least one neurological symptom.^17,18^ These studies have been small, have used self-report from the patient or provider, and have relied on composite outcomes such as HRQOL, and/or reported only absolute rather than relative measures of the outcomes, which can hinder interpretation when study populations vary demographically from each other and from the general population. As far as we are aware, there are no multi-institutional studies of the relative risks of multiple, specific long-term sequelae among persons with a history of NMM.

We hypothesized that meningioma survivors have a greater incidence of neurological and psychological morbidity. To test this, we estimated standardized incidence ratios for neurological and psychological symptoms in a large population with NMM, comparing to a population-based cohort. In doing so, we explore the feasibility of using electronic health records to study these outcomes in larger populations than previously studied. The specific outcomes selected for analysis were based on a review of the literature on long-term sequelae among survivors of CNS tumours of any type. The desired outcome for the research is to inform patients and providers of specific challenges patients with a history of NMM might face and to identify priorities for future research in this area.

## Methods

### Study Design and Data Sources

This was a prospective cohort study using data from the UK Biobank cohort. The UK Biobank is a population-based cohort of approximately half a million people recruited in the United Kingdom between 2006 and 2010. All participants have linked data from hospital records, mortality records, and the cancer registry, and a subset have been linked to primary care data. Specific data sources for each of the participating nations (England, Scotland, and Wales) are provided in Supplementary Table 1.

We identified participants with a new diagnosis of NMM in the cancer registry. Cancer registries collect information on all primary cancers (and some non-malignant neoplasms, such as NMM) diagnosed in individuals primarily resident in their catchment areas. NMMs are usually differentiated from malignant meningiomas based on WHO Grade. As the cancer registry data does not routinely collect WHO Grade, we used morphology/histology and site/topography to identify diagnoses of NMM. NMM diagnoses were identified based on the following codes: ICD-10 - D320: Benign neoplasm of cerebral meninges; D321: Benign neoplasm of spinal meninges; D329: benign neoplasm of meninges, unspecified; ICD-O-3 - 9530/0: Meningioma, NOS; 9531/0; Meningothelial meningioma; 9532/0: Fibrous meningioma; 9533/0: Psammomatous meningioma; 9534/0: Angiomatous meningioma; 9537/0: Transitional meningioma. These may have been radiographically or histologically confirmed cases. We refer to these tumours as non-malignant rather than benign to reflect preferences in the patient and advocacy communities.^19^

We restricted to NMM diagnoses occurring after entry into UK Biobank to reduce the selection bias that could occur if people with pre-existing NMM diagnoses declined to participate in the UK Biobank study. In a supplemental analysis, we extended the “exposed” definition to include people who had been diagnosed with a NMM prior to registration with UK Biobank.

Ten neurological/psychological outcomes were selected for analysis based on findings of a literature review, which suggested increased risks of these among survivors of central nervous system tumours. These were: cognitive deficits/impairment,^8–12,14–18,20–22^ epilepsy,^8,14,17,18,23^ visual impairment or disturbances,^8,9,17,21,23,24^ depression,^10,11,14,20,22^ anxiety,^10,11,14,20,24^ fatigue,^9,14,16,24^ limb weakness,^8,9,24^ hearing loss,^9,21,23^ headache,^17,23^ and stroke.^17,25^ Insomnia^14,16^ and anosmia^23^ were also considered for inclusion but ultimately excluded due to low levels of recording in the electronic health records.

We used outcomes identified using inpatient records from hospital episode statistics. ICD-10 code lists were developed using opencodelists.org and manual searching and are provided in the supplement (Supplementary Table 2). Each hospital encounter can have up to 20 codes so the code did not have to be present as a primary complaint. In our primary analysis, we considered any instance of the code occurring during follow-up. In other words, the outcome did not have to be the first ever instance for the event to be included, since many of the outcomes we were exploring may also occur as symptoms prior to a meningioma diagnosis. In sensitivity analyses, we restricted to the first ever diagnosis or occurrence of the code.

For the cohort at large, follow-up began at the date of assessment for UK Biobank. Participants were excluded if their age at UK Biobank assessment was less than 40 since being aged 40 to 65 was a criterion for UK Biobank entry (*n* = 7). One participant was excluded because their record was missing information on age at assessment and year of birth.

Patients were censored if they died, left the study, or at the end of the study period. At the time of data extract, the end date of the linked data sources varied through 2021. We set the end of follow-up to be March 2, 2021, the latest date an incident NMM was recorded.

For the “exposed” group of NMM patients, follow-up began 6 months after the date of diagnosis. The 6-month latency period was selected in order to reduce the likelihood of including acute/short-term side effects of treatment. Sensitivity analyses explored the impact of eliminating the latency period.

In exploratory analyses we stratified the results by receipt of surgery. A list of OPCS-4 procedure codes designating a meningioma surgery were selected and is provided in the supplement (Supplementary Table 2). We considered the surgery related to the diagnosis if it occurred at most 2 months prior and up to 6 months after diagnosis. Seventy-five percent of all surgeries matching the OPCS-4 procedure codes occurring among meningioma patients occurred within that time window.

### Statistical Analysis

We estimated standardized incidence ratios of each outcome. A standardized incidence ratio is the ratio of the expected rate of events to the observed rate. Expected rates were generated for the UK Biobank as a whole by age (10-year age groups), sex, and calendar period (5 years). Because meningioma cases make up only 0.1% of the cohort, their inclusion in the background rates is considered unlikely to impact the results. Standardized incidence ratios (SIRs) were stratified by age (at the time of follow-up/event) and sex. We reported SIRs for the subgroup of patients who were recorded as having surgery, but since the number of events in patients without surgery was consistently small, and because the definition of surgery within 6 months may have misclassified some people who received surgery later, we have not reported SIRs for persons who were not recorded to have had surgery.

### Patient and Public Involvement

Patients were consulted during 2 rounds of application for funding. At both instances, patients emphasized the usefulness of the work and provided some wording suggestions for the lay abstract. While the analyses were in progress, D.W. presented at 2 meetings of the *brainstrust* Meningioma support group. Patients expressed their gratitude for the research, and relayed experiences of feeling disappointed and frustrated by health care providers who attributed their symptoms to ageing or menopause. Patients also suggested other symptoms to include and/or explore however these were not coded specifically or frequently enough to be included in this study. Following these group meetings, H.B. was invited to be included as a co-author to represent the patient/carer perspective.

### Ethics

UK Biobank has approval from the North West Multi-centre Research Ethics Committee (MREC) as a Research Tissue Bank (RTB) approval. This approval means that researchers do not require separate ethical clearance and can operate under the RTB approval.

## Results

The cohort at large contained just over half a million people (*n* = 501 937) with a mean age at entry to the UK Biobank cohort of 56.5 years (standard deviation [SD]: 8.1) and 11.8 years of follow-up (SD: 1.7, Table 1). Four hundred and sixty-seven NMMs were diagnosed during follow-up (0.1% of the cohort). Persons with NMM joined the UK Biobank at an older age (58.1 years, SD: 7.6) and had less follow-up (5.1 years, SD: 3.0), since follow-up began at the time of their diagnosis. The mean age at NMM diagnosis was 65 (SD: 7.9) years.

**Table 1.** Demographics at UK Biobank Baseline

	No meningioma	Meningioma
	*N* = 501 937	*N* = 467
	Mean (SD)	Mean (SD)
Age at assessment	56.53 (8.10)	58.11 (7.59)
Age at meningioma diagnosis		65.38 (7.89)
Years of follow-up	11.76 (1.72)	5.09 (3.03)
	*n* (%)	*n* (%)
Sex		
Female	272 965 (54.4%)	359 (76.9%)
Male	228 972 (45.6%)	108 (23.1%)
Country at assessment		
England	444 515 (88.6%)	415 (88.9%)
Wales	20 787 (4.1%)	17 (3.6%)
Scotland	36 583 (7.3%)	35 (7.5%)
Ethnicity		
Other/Mixed/Unknown	30 249 (6.0%)	26 (5.6%)
Any white background	471 688 (94.0%)	441 (94.4%)
Townsend Quintile		
5 - Least deprived	229 159 (45.7%)	227 (48.6%)
4	110 739 (22.1%)	100 (21.4%)
3	73 430 (14.6%)	68 (14.6%)
2	60 146 (12.0%)	51 (10.9%)
1 - Most deprived	27 837 (5.6%)	21 (4.5%)
Smoking status		
Never	273 209 (54.4%)	262 (56.1%)
Previous	172 851 (34.4%)	172 (36.8%)
Current	52 932 (10.5%)	29 (6.2%)
Missing/unknown	2 945 (0.6%)	4 (0.9%)
Body mass index (BMI)		
Underweight	2 626 (0.5%)	0 (0.0%)
Healthy	162 219 (32.3%)	149 (31.9%)
Overweight/Obese	333 988 (66.5%)	315 (67.5%)
Missing	3 104 (0.6%)	3 (0.6%)

Patients with a history of NMM were more likely to be female than the cohort as a whole (77% vs. 54%, Table 1). Those who would be diagnosed with NMM were not notably different from the cohort with respect to ethnicity (White vs. other, mixed, or unknown), deprivation quintile, or body mass index at enrolment to the UK Biobank. NMM patients were less likely to smoke at baseline than the cohort as a whole. This was the case even after accounting for sex (8.9% among cohort vs. 5% among NMM in females and 12.5% vs. 10.2% in males).

**Figure 1.**
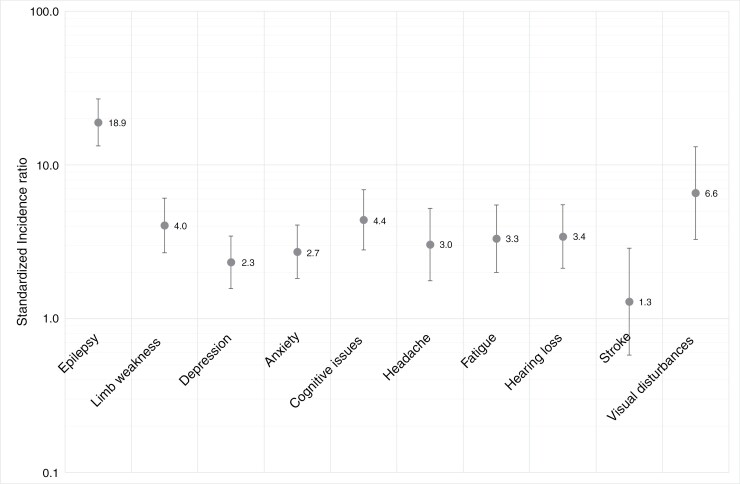
Standardised Incidence Ratios (SIR)s and 95% Confidence Intervals for NMM Cases Compared to the UK Biobank as a Whole.

As shown in Figure 1 and Table 2, NMM patients were significantly more likely to experience 9 out of 10 of the neurologic outcomes under study. The SIR was highest for epilepsy, where NMM patients had incidence 18.9 times higher than the cohort overall (95% confidence interval [CI]: 13.3-26.9). The lowest SIR and the only one that was non-significant was for stroke (1.3, 95% CI: 0.6-2.9). All other SIRs ranged between 2.3 (depression, 95% CI: 1.6-3.4) and 4.4 (cognitive issues, 95% CI: 2.8-6.9).

**Table 2.** Standardised Incidence Ratios (SIRs) and 95% Confidence Intervals (CI) for Each Outcome, Stratified by Surgical Receipt, Age^a^, and Sex.^b^

	*n*	Observed	Expected	SIR	95% CI
Epilepsy					
Overall	467	31	1.6	18.9	(13.3, 26.9)
With surgery	333	29	1.2	24.7	(17.2, 35.6)
<70 years of age^a^		19	0.8	25.2	(16.1, 39.5)
≥70 years of age		12	0.9	13.5	(7.7, 23.8)
Female	363	19	1.2	15.4	(9.8, 24.1)
Male	115	12	0.4	29.6	(16.8, 52.2)
Limb weakness					
Overall	467	23	5.7	4.0	(2.7, 6.1)
With surgery	333	16	4.0	4.0	(2.5, 6.6)
<70 years of age^a^		9	1.5	5.9	(3.1, 11.4)
≥70 years of age		14	4.2	3.4	(2.0, 5.7)
Female	363	14	4.3	3.3	(1.9, 5.5)
Male	115	9	1.4	6.3	(3.3, 12.1)
Depression					
Overall	467	25	10.7	2.3	(1.6, 3.4)
With surgery	333	17	7.9	2.2	(1.3, 3.5)
<70 years of age^a^		16	5.5	2.9	(1.8, 4.7)
≥70 years of age		9	5.2	1.7	(0.9, 3.3)
Female	363	18	9.1	2.0	(1.3, 3.2)
Male	115	7	1.7	4.2	(2.0, 8.8)
Anxiety					
Overall	467	24	8.8	2.7	(1.8, 4.1)
With surgery	333	20	6.2	3.2	(2.1, 5.0)
<70 years of age^a^		14	3.8	3.7	(2.2, 6.2)
≥70 years of age		10	5.0	2.0	(1.1, 3.7)
Female	363	19	7.5	2.5	(1.6, 3.9)
Male^b^					
Cognitive issues					
Overall	467	19	4.3	4.4	(2.8, 6.9)
With surgery	333	12	3.0	4.0	(2.3, 7.0)
<70 years of age^a^		7	1.1	6.4	(3.0, 13.4)
≥70 years of age		12	3.2	3.7	(2.1, 6.6)
Female	363	15	3.0	2.0	(3.0, 8.2)
Male^b^					
Headache					
Overall	467	13	4.3	3.0	(1.8, 5.2)
With surgery	333	10	3.1	3.2	(1.7, 6.0)
<70 years of age^a^		8	2.1	3.9	(1.9, 7.7)
≥70 years of age					
Female	363	12	3.6	3.4	(1.9, 5.9)
Male^b^					
Fatigue					
Overall	467	15	4.5	3.3	(2.0, 5.5)
With surgery	333	9	3.1	2.9	(1.5, 5.5)
<70 years of age^a^					
≥70 years of age		12	3.4	3.6	(2.0, 6.3)
Female	363	10	3.4	2.9	(1.6, 5.5)
Male^b^					
Hearing loss					
Overall	467	17	5.0	3.4	(2.1, 5.5)
With surgery	333	13	3.5	3.7	(2.2, 6.4)
<70 years of age^a^		10	1.4	7.2	(3.9, 13.3)
≥70 years of age		7	3.6	2.0	(0.9, 4.1)
Female	363	16	3.5	4.6	(2.8, 7.4)
Male^b^					
Stroke					
Overall	467	6	4.7	1.3	(0.6, 2.9)
With surgery^b^					
<70 years of age^a^					
≥70 years of age					
Female^b^					
Male^b^					
Visual disturbance					
Overall	467	8	1.2	6.6	(3.3, 13.1)
With surgery	333	6	0.9	7.0	(3.1, 15.5)
<70 years of age^a^					
≥70 years of age					
Female	363	8	0.9	9.0	(4.5, 17.9)
Male^b^					
				

^a^Numbers of individuals eligible are not provided for subcategories since patients move between categories of age during follow-up.

^b^Results omitted when based on ≤5 observed events.

Stratified analyses are presented in Table 2. Stratified SIRs were not presented when based on fewer than 5 events, so have been omitted for several outcome/subgroups. When reported, SIRs tended to be similar in the subgroup undergoing surgery, tended to be higher in those aged less than 70 than those aged older than 70, and tended to be higher in males than in females.

As described in the Methods section, we conducted 2 sensitivity analyses: (1) restricting to first ever occurrence of an outcome code as an event, (2) removing the latency period. We also did a supplemental analysis including prevalent (existing) and incident (new) meningioma diagnoses. Overall, after considering uncertainties, the results from all 4 analyses are very similar (Supplementary Table 3). As might be expected, SIRs tended to be marginally higher when the latency period was removed and marginally lower when prevalent diagnoses (those diagnosed prior to cohort entry) were included. When the latency period for stroke was removed, the SIR increased and was statistically significant (SIR: 2.4, 95%CI: 1.4-4.2).

## Discussion

This is the first study that we are aware of to examine long-term outcomes among individuals diagnosed with NMM using linkage to electronic health records. This approach allowed us to efficiently follow over 450 patients for a median of 5 years. We observed significantly increased risk of 9 out of 10 conditions examined, with these conditions being between twice and 20 times more common among persons with NMM than background rates in the cohort. This evidence can serve to validate the lived experience of NMM patients and improve patient communication about the outcomes of treatment for NMM. Furthermore, this study serves as a proof-of-concept and evidence that further research, using larger datasets that include primary care records, which would capture a broader range of sequelae with more sensitivity than hospital records, would be valuable.

As most of the studies to date have been survey-based, have included survivors of other types of brain tumours, and/or have not had estimates of relative risk, it is difficult to compare our findings quantitatively to existing published work. We chose our list of outcomes based on the literature, so insofar as we saw an increased risk of these factors in our cohort, our findings are consistent.^8–10,12,13^ The standardized incidence ratio was highest for seizures/epilepsy, which may be at least in part attributable to the low background rate in the population relative to the other outcomes examined. Stroke was the only outcome that was not significantly elevated in NMM patients, despite being suggested by our literature review as a potential risk. This may be because stroke is likely to occur peri-operatively. When we removed the 6-month latency period in the sensitivity analyses, there was a significantly increased risk of stroke among people with a history of NMM.

The study has 4 primary strengths. First, using incident cases of NMM from within an existing population-based cohort reduced the risk of selection bias, whereas enrollees to a cohort study with a prior history of NMM may not be representative persons with a diagnosis of NMM overall. Second, using administrative health records lends an objectivity to the ascertainment of outcomes. Cognitive decline experienced by patients, for example, may reduce the accuracy of self-reporting of outcomes. Third, the use of administrative health records allowed us to conduct the largest study of specific long-term outcomes in NMM of which we are aware. Fourth, this approach allowed us to achieve a median follow-up of 5 years among cases relatively efficiently, with very high follow-up and low rate of attrition. Finally, because SIRs compare observed to expected rates, they also account for differences in follow-up time between the two comparison groups.

There are, however, also several limitations to this analysis. We have tried to address some of these using the sensitivity analyses described above. One major limitation is that we used hospital, rather than primary care records. In the initial design of the study, we anticipated more primary care coverage and/or an update of the UK Biobank primary care dataset during the project. At the time of data release, however, primary care linkage was available for approximately 45% of the cohort, with data spanning up until 2016 or 2017. Restricting to those who had primary care data reduced the number of NMM patients by two-thirds, leading to a loss in power that outweighed advantages in sensitivity provided by the primary care records. We also explored using follow-up surveys in the UK Biobank for self-reported follow-up but the number and timing of responses was insufficient to provide meaningful additional data. In the present study, diagnosis codes in hospital records alone were used to assess all outcomes/sequelae.

Some of our outcomes would not merit admission to hospital. While this is true for both NMM cases and comparators, because the event needs to be recorded in the hospital record, increased risks may simply reflect greater contacts with the secondary care system among NMM cases. Due to the reliance on hospital records, the count of events is presumed to highly underestimate the true incidence of any given condition. The extent to which the hospital recording of a diagnosis or symptom represents the true occurrence will depend on the importance of the diagnosis to treatment and/or hospital processes and reimbursement. As a result, a stroke will be more likely to be recorded than a visual disturbance, for example. To address this in part, we have focussed on ratios and intentionally not reported rates. Nevertheless, we acknowledge this as a major limitation and call for studies using primary care data in larger populations.

Furthermore, UK Biobank participants are healthier, more affluent, and more likely to be white than members of the UK population at large.^26^ While background rates of morbidity will be underestimated in the UK Biobank cohort relative to the general population, we believe the SIRs could be internally valid since people diagnosed with NMM after entry into the UK Biobank cohort will have been subject to the same “healthy volunteer bias.” The cancer data linked to the UK Biobank cohort did not include information on tumour location, method of confirmation (eg radiological or histological), recurrence, or aspects of treatment such as radiotherapy and/or whether the resection was complete or partial. Studies with this information could generate more informative, stratified risk estimates. Finally, while this study constitutes one of the largest cohorts of NMM patients followed longitudinally, the use of SIRs rather than causal modelling and relative risks reflects the descriptive nature of the research and the small sample size for certain outcomes (ie insufficient power to control for numerous covariates).

Despite the significant limitations to this study, our results complement survey data reporting deficits in quality of life and decreased participation in work, social, and recreational activities among persons with a history of NMM.^15^ Together, they suggest that these patients would benefit from care that reflects their increased risks of psychological and neurological late effects. In a recent study of unmet needs in people with a diagnosis of NMM, information on possible long-term complications, expectation management, and research on outcomes were all identified as high importance.^25^ In combination with evidence from other sources, our findings could be integrated into materials for patients, including survivorship care plans and into clinical conversations with patients, before, during and after treatment.

## Supplementary Material

vdaf105_suppl_Supplementary_Materials

## Data Availability

The datasets generated by UK Biobank analysed during the current study are available via the
